# Dosimetric consequences of image guidance techniques on robust optimized intensity-modulated proton therapy for treatment of breast Cancer

**DOI:** 10.1186/s13014-020-01495-6

**Published:** 2020-02-27

**Authors:** Xiaoying Liang, Raymond B. Mailhot Vega, Zuofeng Li, Dandan Zheng, Nancy Mendenhall, Julie A. Bradley

**Affiliations:** 1 Department of Radiation Oncology, University of Florida College of Medicine, Jacksonville, FL USA; 2 Department of Radiation Oncology, University of Nebraska Medical Center, Omaha, NE USA

**Keywords:** Breast cancer, Intensity-modulated proton therapy, Dosimetric consequences, Alignment techniques

## Abstract

**Purpose:**

To investigate the consequences of residual setup error on target dose distribution using various image registration strategies for breast cancer treated with intensity-modulated proton therapy (IMPT).

**Materials and methods:**

Among 11 post-lumpectomy patients who received IMPT, 44 dose distributions were computed. For each patient, the original plan (Plan-O) and three verification plans were calculated using different alignments: bony anatomy (VPlan-B), breast tissue (VPlan-T), and skin (VPlan-S). The target coverage were evaluated for each alignment technique. Additionally, 2 subvolumes—BreastNearSkin (1-cm rim of anterior CTV) and BreastNearCW (1-cm rim of posterior CTV)—were created to help localize CTV underdosing. Furthermore, we divided the setup error into the posture error and breast error. Patients with a large posture error and those with good posture setup but a large breast error were identified to evaluate the effect of posture error and breast error.

**Results:**

For Plan-O, VPlan-B, VPlan-T, and VPlan-S, respectively, the median (interquartile range) breast CTV D95 was 95.7%(94.7–96.3%), 95.1% (93.9–95.7%), 95.2% (94.8–95.6%), and 95.2% (94.9–95.7%); BreastNearCW D95 was 96.9% (95.6–97.3%), 94.8% (93.5–97.0%), 95.6% (94.8–97.0%), 95.6% (94.8–97.1%); and BreastNearSkin D95 was 94.1% (92.7–94.4%), 93.6% (92.2–94.5%), 93.5% (92.4–94.5%), and 94.4% (92.2–94.5%) of the prescription dose. 4/11 patients had ≥1% decrease in breast CTV D95, 1 of whom developed breast edema while the other 3 all had a > 2^o^ posture error. The CTV D95 variation was within 1% for the patients with good posture setup but >2^o^ breast error.

**Conclusion:**

Acceptable target coverage was achieved with all three alignment strategies. Breast tissue and skin alignment maintained the breast target coverage marginally better than bony alignment, with which the posterior CTV along the chest wall is the predominant area affected by under-dosing. For target dose distribution, posture error appears more influential than breast error.

## Introduction

Radiotherapy plays a significant role in the treatment and cure of breast cancer. However, conventional photon-based radiotherapy, while effective, is also associated with increased risks of cardiovascular morbidity and mortality due to incidental radiation to the heart. Darby et al. [1] reported that the incidental exposure of the heart to radiation in the treatment of breast cancer increases the rate of major coronary events by 7.4% per Gy, with no apparent threshold. Due to its unique energy deposition profile, proton therapy can reduce the volume of the heart and lungs exposed to radiation, thereby potentially reducing the side-effect [2–8]. Therefore, in the past few years, there is an increasing interest in the use of proton therapy in the treatment of breast cancer [9, 10]. While proton therapy offers significant dosimetric advantages for sparing organs at risk (OARs), it is also sensitive to setup errors. The dose distribution, which is the result of multiple pristine Bragg peaks of energy deposition, can be altered by variations in water equivalent thickness (WET) of the beam paths associated with setup uncertainty and/or organ motion.

The positioning of patients with breast cancer can be challenging due to both the large target volume and the highly mobile breast tissue. Michalski et al. [11] conducted a systematic review of the literature on inter- and intra-fraction motion in photon therapy for breast cancer. They reported an average motion below 5 mm, but observed up to 20 mm in deviation for some patients. Batumalai et al. [12] reviewed the literature on setup errors in photon therapy focusing on supine breast radiotherapy using cone-beam computed tomography (CBCT) and reported up to 5.7 mm, 3.8 mm, 5.7 mm systematic errors and 7.3 m, 4.1 mm, 4.0 mm random errors in in the left-right, superior-inferior, and anterior-posterior direction, respectively.

Image-guided radiation therapy (IGRT) helps to address inter-fraction motion and improves the precision of treatment delivery. IGRT techniques have rapidly evolved for photon therapy, but in proton therapy they have remained relatively behind. Setup on bony structures on 2-dimensional images using digital radiography is currently the standard technology at proton centers. In recent years, IGRT techniques have been gradually catching up in proton therapy. CBCT has become available at some proton centers [13, 14] and the use of surface imaging [15] has also been reported. Despite advanced alignment techniques, it is practically impossible to have a perfect alignment on every voxel, and residual setup errors persist. To date, the consequence of the residual setup error on dose distribution for proton therapy in the treatment of breast cancer has not been reported. Many important questions remain unanswered: What is the dose error attributable to the relative motion between the breast tissue and the bony anatomy when the patient is aligned using bony structures? And which image guidance technique yields the least residual setup error so that the delivered dose is best maintained as planned? To answer these questions, we designed and conducted the current study.

## Methods and materials

### Patient selection

This study was approved by our institutional review board (IRB# 201702651). This study included 11 female post-lumpectomy patients consecutively treated with intensity-modulated proton therapy (IMPT). Of these, 9 patients received IMPT to the whole breast and regional lymph nodes including internal mammary nodes (IMN), axillary level I-III nodes (AxI-III), and supraclavicular nodes (SCV). 2 received IMPT to only the breast. As per standard of care at our center, all patients underwent a repeat simulation CT (thereafter referred to as verification CT) about halfway through the treatment course to evaluate breast changes (owing to breast edema, change in seroma, etc.) and setup reproducibility, and confirm stable dosimetry. The breast volume on the planning CT and the breast volume variation on the verification CT are shown in Table 1. The treatment sites are also shown.

**Table 1:** Patient characteristics

Patient #	Breast tissue volume on the planning CT cm^3^	Breast tissue volume variation on the verification CT (%)	Treatment site
1	958.1	4.8	Lt Breast only
2	372.3	−1.1	Lt Breast+LNs
3	742.2	−4.5	Lt Breast+LNs
4	858.4	1.8	Lt Breast only
5	1537.7	−4.2	Rt Breast+LNs
6	1505.1	−0.5	Lt Breast+LNs
7	991.4	7.9	Rt Breast+LNs
8	1067.0	−4.1	Rt Breast+LNs
9	949.2	−6.1	Lt Breast+LNs
10	1780.4	1.8	Lt Breast+LNs
11	1361.7	−1.7	Lt Breast+LNs

Abbreviations: *Lt* left; *Rt* Right; *LNs* lymph nodes

### Treatment simulation, segmentation, and planning

Patients were simulated using a Philips Brilliance Big Bore CT (Philips Healthcare, The Netherlands) in the supine position with arms above their heads using a CIVCO breast board (CIVCO Radiotherapy, Coralville, IA, USA). Four-dimensional computed tomography (4DCT) scans were acquired, from which the average-intensity-projection CT was used for treatment planning and for generating digitally reconstructed radiography (DRR). The DRR generated from the average CT is considered a more realistic reference for daily setup given the movement of the chest wall with respiration.

The clinical target volume (CTV) structures, including breast tissue limited anteriorly 5 mm from the skin (CTVbreast), IMN, AxI-III, and SCV, were contoured. OARs, including the heart, left anterior descending artery (LAD), left lung, right lung, esophagus, and thyroid, were also contoured. Along the CTVs, a layer of skin structure 5 mm inward from the body (skin5mm rim) was contoured as well.

Treatment planning was conducted on a RayStation treatment planning system (RaySearch Laboratories, Sweden) (V6.1). The dose prescription was 50 GyRBE in 25 fractions. For each plan, 2 en-face beam angles were used. A water-equivalent 7.4-cm Lucite range shifter was used for each beam. Robust optimization was utilized on each of the CTV structure with 5 mm setup uncertainty and 3.5% range uncertainty. The 5 mm setup uncertainty and 3.5% range uncertainty robust optimization parameters were based on existing literatures [11, 12, 16]as well as our clinical experience. Both the optimization and dose computation used a Monte Carlo algorithm. The plans were normalized so that 92–95% of the target volume received 95% of the prescription dose according to the treating physician’s discretion. For planning goals, ideal target coverage of CTV was V95% ≥ 95% and D95% ≥ 95%, but V90% ≥ 90% and D90% ≥ 90 were considered acceptable.

### Evaluation of the impact of patient setup techniques

To simulate patient setup during treatment [1] on bony structures using orthogonal KV imaging, [2] on soft tissue using CBCT, and [3] on skin surfaces using surface imaging, the verification CT was registered to the planning CT through alignment with [1] bony structures, [2] breast tissue, and [3] skin (skin5mm rim), respectively. All image registrations were performed in RayStation using automatic image registration on the specified regions of interest (ROIs) as detailed below, eliminating operator bias. The original plan was calculated on the verification CT and the dose distributions on the verification CT with different registration strategies were compared with the planned doses to determine the impact of the different patient setup techniques.

For each patient, 4 dose distributions were calculated:

1. Original plan (Plan-O), that is, the clinical plan used for patient treatment.
2. Verification plan using bony alignment (VPlan-B) wherein the verification CT was rigidly registered to the planning CT on the bony anatomy.
3. Verification plan using breast tissue alignment (VPlan-T) wherein the verification CT was rigidly registered on the breast tissue.
4. Verification plan using skin alignment (VPlan-S) wherein the verification CT was rigidly registered on the skin5mm rim.

For each verification plan, the verification dose was obtained by compute the original plan on the verification CT, using the same settings—such as pencil-beam scanning (PBS) energy layers, spot geometry and weighting, and monitor units—as those in the original plan.

The target coverage on CTVbreast was evaluated using V90, V95, D90, D95, and D99. In addition, two subvolumes—BreastNearSkin and BreastNearCW—were delineated to enable localization of specific areas of under-dosing. The BreastNearSkin and BreastNearCW were constructed by taking a 1-cm rim of the CTVbreast volume along the skin and along the chest wall, respectively. These two subvolumes were evaluated on V90, V95, D90, and D95. The D90 and D95 on the regional lymph nodes were also evaluated. Pairwise differences in the evaluated dosimetric metrics between the original plan and each of the verification plans were evaluated using a non-parametric Wilcoxon signed rank test with *p* < 0.05 taken as significant.

### Evaluation of setup errors

We divided the setup error into [1] “posture error” refers to general posture setup inaccuracies, such as pitch, roll and rotation and [2] “breast error” refers to the breast rotations and shape changes with respect to the patient’s bony structures. Posture errors is measured by bony anatomy difference (pitch, roll and rotation) between the verification CT and the reference (planning) CT via rigid registration using the bony structures. Breast errors can be assessed through the rotational difference between registration using the breast tissue and bony anatomy. To study the effect of posture errors and breast errors separately, we identified patients with a large posture error and those with good posture setup but a large breast error. The dose error of these two subgroups were evaluated. In the current study, we defined cases with >2^o^ rotation in any direction as a large error.

## Results

### Target coverage with the different alignment strategies

Variations in target volume delineation for breast treatment have been studied [17, 18] and Hurkmans et al. [18] reported a 5.5% (standard deviation) in intra-observer variation, which aligns with our clinical experience. When re-contouring the CTVs on the verification CT, we considered that the contouring variation would contribute ≤5% of the volume variation. Therefore, for cases in which the breast volume variation > 5%, we further assessed these as special cases and sought a cause. We found 2 cases with > 5% breast volume variations, patients #7 (7.9%) and #9 (− 6.1%), were attributed to breast edema and weight loss during the treatment, respectively. No such changes were observed in the other 9 patients. Therefore, we present our data in two groups: the first group includes the 9 patients with breast volume variations within 5%; the second group includes the 2 special cases where edema or weight loss occurred during the treatment. For these special cases, replanning is indicated regardless of the selected alignment strategy.

### Target dose coverage with stable breast volume (*n* = 9)

**Fig. 1:**
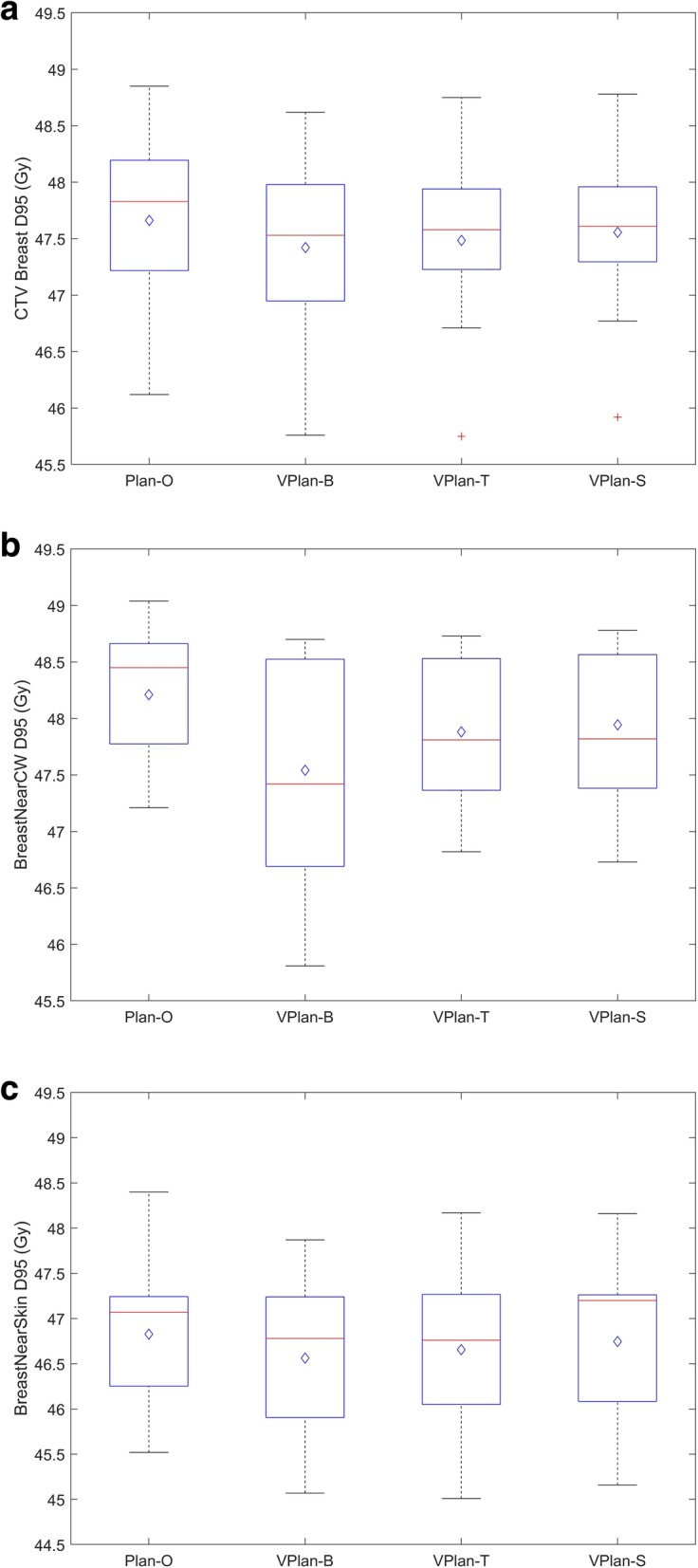
Box plot of CTVbreast D95, BreastNearCW D95, and BreastNearSkin D95. The red line inside the box represents the median value and the blue diamond represents the mean value

We first evaluated the V90 and D90 on each patient with each type of alignment technique and found that our institutional goals of CTV V90 ≥ 90% and CTV D90 ≥ 90% (defined as acceptable) were met in all cases. Table 2 lists the dose statistics for CTVbreast and the two subvolume structures, BreastNearSkin and BreastNearCW. For better visualization, Fig. 1 shows the box plot on the D95 of CTVbreast, BreastNearCW, and BreastNearSkin. In general, alignment with the breast tissue and the skin marginally better preserved the CTVbreast dose compared to alignment with bony structures, with which the posterior CTV along the chest wall is the predominant area affected by under-dosing. None of the alignment strategies yielded a statistically significant difference in target dose coverage from the original plan.

**Table 2:** Median (IQR) of the CTV Breast, BreastNearSkin, BreastNearCW, and CTV nodal coverage. The dose is displayed in percentage of the prescription dose

Dosimetric parameters	Plan-O		Vplan-B		VPlan-T		VPlan-S		*P* values		
	Med	IQR	Med	IQR	Med	IQR	Med	IQR	Plan-O vs. Vplan-B	Plan-O vs. Vplan-T	Plan-O vs. Vplan-S
CTVbreast V90 (%)	99.7	99.3–99.8	98.8	98.7–99.6	99.6	98.6–99.6	99.6	98.6–99.8	0.22	0.34	0.60
CTVbreast V95 (%)	96.4	93.32–97.33	95.1	91.7–96.3	95.2	94.1–97.0	95.3	94.7–97.0	0.55	0.60	0.67
CTVbreast D90 (%)	96.5	95.4–97.5	96.3	95.3–97.5	96.4	95.6–97.3	96.4	95.7–97.5	0.75	0.80	0.93
CTVbreast D95 (%)	95.7	94.7–96.3	95.1	93.9–95.7	95.2	94.8–95.6;	95.2	94.9–95.7	0.22	0.45	0.55
CTV Breast D99 (%)	92.6	90.8–92.8	89.2	88.6–91.6	91.8	88.5–92.7	91.8	88.5–93.0	0.60	0.55	0.62
BreastNearCW V90 (%)	99.6	99.0–99.9	99.0	97.3–100	98.9	98.3–100	99.1	97.8–100	0.59	0.47	0.62
BreastNearCW V95 (%)	98.3	97.7–99.5	94.6	93.4–99.7	97.0	94.6–99.7	97.1	94.7–99.8	0.49	0.67	0.80
BreastNearCW D90 (%)	97.4	96.0–97.8	96.5	95.9–97.6	97.3	95.9–97.7	97.5	95.9–97.8	0.34	0.39	0.86
BreastNearCW D95 (%)	96.9	95.6–97.3	94.8	93.5–97.0	95.6	94.8–97.0	95.6	94.8–97.1	0.18	0.27	0.45
BreastNearSkin V90 (%)	99.4	98.5–99.5	98.7	98.3–99.5	98.9	97.2–99.6	99.1	97.2–99.6	0.49	0.95	0.84
BreastNearSkin V95 (%)	91.0	84.9–93.1	91.3	83.5–93.7	92.3	86.0–93.3	92.8	85.9–93.4	1.00	0.93	0.93
BreastNearSkin D90 (%)	95.5	94.1–96.0	95.2	93.6–96.4	95.5	94.2–95.7	95.2	94.2–96.0	1.00	0.81	0.98
BreastNearSkin D95 (%)	94.1	92.7–94.4	93.6	92.2–94.5	93.5	92.4–94.5	94.4	92.2–94.5	0.62	0.93	0.85
IMN D90 (%)	96.8	96.6–97.4	96.5	95.4–97.2	96.6	95.0–97.1	96.5	94.8–96.9	0.40	0.54	0.32
IMN D95 (%)	96.1	95.8–96.7	95.7	94.6–96.4	95.6	94.0–96.6	95.6	93.5–96.3	0.48	0.48	0.26
AXI D90 (%)	96.6	95.0–98.1	96.5	94.9–98.0	96.5	94.9–98.0	96.5	94.9–97.8	0.71	0.80	0.93
AXI D95 (%)	96.2	94.2–97.8	96.1	94.1–97.7	96.2	94.1–97.7	96.2	94.1–97.4	0.64	0.80	0.80
AXII D90 (%)	96.0	93.4–97.3	95.9	93.1–97.3	96.0	93.2–97.3	96.0	93.3–97.1	0.80	0.90	0.98
AXII D95 (%)	95.6	92.3–96.9	95.4	92.6–96.9	95.5	92.8–96.9	95.5	92.8–96.5	0.90	0.90	0.90
AXIII D90 (%)	96.4	93.6–97.1	96.3	93.7–97.0	96.1	94.0–97.0	96.2	93.8–97.0	0.80	0.71	0.90
AXIII D95 (%)	95.9	92.9–96.7	95.8	93.1–96.6	95.6	93.1–96.5	95.6	93.2–96.5	0.80	0.83	0.90
SCV D90 (%)	96.9	93.6–97.5	96.7	93.9–97.3	96.6	94.1–97.3	96.7	94.2–97.3	0.78	0.93	0.74
SCV D95 (%)	96.5	92.5–96.9	96.3	93.1–96.7	96.1	93.2–96.7	96.3	93.3–96.7	0.80	0.90	0.80

Abbreviation: *Med* median; *IQR* interquartile range; BreastNearSkin, 1-cm rim of anterior CTV; BreastNearCW, 1-cm rim of posterior CTV

For dose coverage of the regional lymph nodes, all three alignment strategies were able to meet our institutional acceptability criteria. Table 2 also lists the dose statistics on the nodal volumes. Patients #1 and #4 were treated on the breast only. Therefore, the data on lymph nodes includes 7 patients. The three alignment strategies yielded comparable regional lymphatics coverage and none of the alignment strategies yielded a statistically significant dose difference from the original plan.

### Target dose coverage with varied breast volume (*n* = 2)

Patient #7 developed breast edema during treatment and had a 7.9% breast tissue volume increase on the verification CT. Because of the increased WET due to the edema, the CTVbreast D95 dropped by 0.8, 1.0, and 1.2% with alignment to the bony structure, breast tissue, and skin, respectively. As expected, the BreastNearCW had the largest decrease in D95: 5.8, 4.7, and 5.0% with alignment using bony structure, breast tissue, and skin, respectively. A re-plan was performed to account for the edema owing to under-coverage near the chest wall.

Patient #9 lost weight during treatment, resulting in a 6.1% breast tissue volume decrease. Despite the breast tissue volume decrease, counter-intuitively, a decrease in dose coverage was observed on CTVbreast D95: 1.3, 0.6, and 0.4% decrease with alignment to bony structure, breast tissue, and skin, respectively. This patient had a 3.5^o^ posture error and the breast error was within 2^o^, indicating that large posture error is prone to under-dosing the target. A re-plan was conducted for this patient due to the potential over-dose to the lung with the decreased breast volume.

### Posture errors and breast errors

Table 3 shows the posture errors and the breast errors. The direction of the rotation was not indicated here; for example, we did not distinguish pitch-up from pitch-down, but chose to focus on the degree of the pitch, regardless of direction.

**Table 3:** Posture errors and breast errors of the 11 studied patients

Patient #	Posture errors			Breast errors		
	Pitch (^o^)	Roll (^o^)	Yaw (^o^)	Pitch (^o^)	Roll (^o^)	Yaw (^o^)
1	0.5	6.3	1.5	1.2	0.8	3.0
2	0.5	0.2	1.1	0.6	0.2	1.4
3	0.1	0.2	0.2	0.6	0.7	0.5
4	0	2.1	1.3	0.7	2.8	1.8
5	1.4	0.1	0.3	0	1.0	0.1
6	0.2	1.9	1.2	0.5	2.6	0.5
7	0.4	0.2	0.7	0.6	1.6	0.1
8	0.8	1.0	1.9	1.1	1.4	2.4
9	1.6	3.5	0.6	0	1.8	0.9
10	2.1	2.2	0.6	0.5	2.2	1.0
11	0	0	0	0.8	4.0	0.7

Among the 11 patients, 4 (patients #1, #4, #9, and #10) showed a > 2^o^ posture error, mainly in the roll rotations. In addition, 6 patients (#1, #4, #6, #8, #10, and #11) showed a > 2^o^ breast error. A large posture error tends to amplify the breast error, especially for pendulous breast. Patients #6, #8 and #11 had reasonable posture setup but a large breast error; therefore, they are good candidates for studying the effect of breast error alone.

**Fig. 2:**
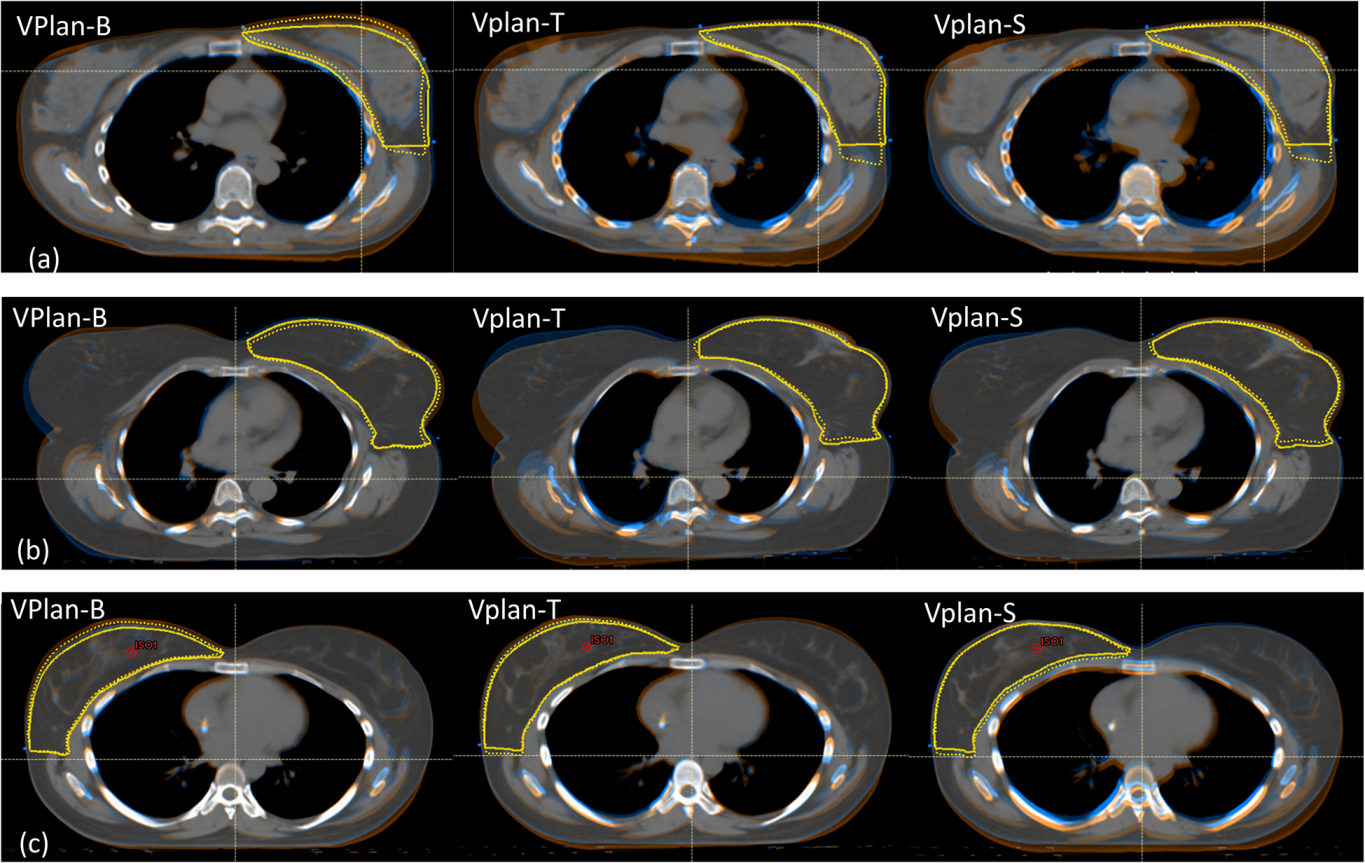
Example of image registration between the verification scan (orange) and the original planning scan (blue) for (**a**) Patient #4, who had a large posture error and breast error, (**b**) Patient #11, who had good posture setup but a large breast error, and (**c**) Patient #7, who developed breast edema during the treatment. The blue or orange color only appears in regions of misalignment. The solid yellow contour represents the CTVbreast contour on the original scan and the dashed yellow contour represents the CTVbreast on the verification scan

Figure 2 shows the image registration of the verification CT (orange) with the planning CT (blue). Figure 2a (Patient #4) had a counter-clock wise roll posture error so that the alignment on bony structures required a clock-wise roll correction, as illustrated in VPlan-B. Due to the counter-clockwise roll posture error, gravity created a breast tissue roll counter-clockwise, to a degree larger than the posture error alone. Therefore, a more clockwise roll correction was required when aligning with the breast tissue, as shown in VPlan-T. The alignment on skin (VPlan-S) showed an overall similar alignment as VPlan-T, but with slightly better alignment on the CTVbreast near the skin and slightly worse alignment on the CTVbreast near the chest wall. Figure 2b (Patient #11) had a good posture setup. However, due to the large (1361.7cm^3^) and pendulous breast, the breast shape changed, requiring a counter clockwise roll to align the breast tissue (VPlan-T) and the skin (VPlan-S). The image registration for the patient who developed edema during treatment is shown in Fig. 2c (Patient #7); an increased WET along the beam path was observed.

To study the effect of posture errors and breast errors, we examined the cases in which the CTVbreast D95 decreased by ≥1% of the prescription dose. In total, 4 of the 11 patients experienced ≥1% decrease in CTVbreast D95: Patients #1 (1.7% with bony alignment), #4 (1.1% with both breast tissue and skin alignments), #7 (1.0% with breast tissue and 1.2% with skin alignments), and #9 (1.3% with bony alignment). As Patient #7 developed breast edema, which caused a WET increase and consequently target coverage loss, we removed her from this particular analysis. Although Patient #9 was also a special case with weight loss, unlike Patient #7, Patient #9 had a breast tissue volume decrease and in principle WET decrease, and was therefore included in this analysis.

We investigated the impact of the breast error by identifying cases that had <2^o^ setup error but >2^o^ breast error (patients #6, #8 and #11). These three cases represent scenarios in which the patient had a reasonable general posture setup, but large breast error. All of these patients had relative large breast volumes (1505.1, 1067.0, and 1361.7 cm^3^), increasing the likelihood of a change in breast shape due to the mobility of the breast tissue. However, none of these patients demonstrated a ≥ 1% decrease in CTVbreast D95 with any alignment strategies, indicating that, with our method of robust optimization, the target coverage is well maintained (within 1% variation) with any type of alignment (bony, breast tissue, skin) even for cases with large breast errors, as long as the patient’s posture setup is good.

We also analyzed the cases (#1, #4, #9, and #10) in which the posture error was large (>2^o^). Among these 4 cases, 3 demonstrated a ≥ 1% decrease in CTVbreast D95 with ≥1 of the alignment strategies. This, together with the fact that the patients who experienced a ≥ 1% decrease in CTVbreast D95 all had a large posture error (with the exception of the breast edema case), indicate that a large posture error tend to have a higher likelihood of decrease the target coverage. Alignment with the breast tissue or with the skin does not guarantee < 1% CTV D95 variation for cases with large posture errors.

## Discussion

Setup for breast patients is challenging and the dosimetric consequences for photon therapy have been well-studied [19–23], but comparable data for proton therapy are scarce. Due to the distinctly different physical properties of photons and protons, and their different treatment planning techniques, experiences with photon therapy cannot be directly translated and applied to proton therapy. The maintenance of target coverage for patients with breast cancer in the setting of inter-fraction motion has not yet been well-studied for proton therapy. The optimal approach for setup breast patients undergoing treatment with proton therapy has not been established. Through this study, we sought to determine [1] the dosimetric consequence of different alignment techniques and [2] the optimal patient setup strategy for breast patients treated with proton therapy.

We studied 11 patients with a median CTVbreast volume of 991.4 cm^3^ (range, 372.3–1780.4 cm^3^) and investigated the dosimetric impact of different alignment techniques on the target coverage for patients with breast cancer undergoing treatment using IMPT. On-treatment verification CT were registered to the planning CT using different alignment techniques: bony structure to simulate setup using orthogonal KV images, breast tissue to simulate setup using CBCT, and skin to simulate setup using surface imaging. Our study showed that our treatment planning technique is robust; acceptable target coverage was maintained using any of these three alignment strategies. Due to the relative motion between the bony structure and breast tissue, we found that the CTV along the chest wall is most susceptible to underdosing when bony structures are used for alignment. Therefore, for patients with a high-risk area near the chest wall, particular attention to the setup error is critical. In this study, we divided the setup error into posture error and breast error to investigate which error would have a greater impact on target dose coverage. We found that the posture error had a greater effect than the breast error. Patients who have a pendulous breast tend to demonstrate larger breast error, as the reproducibility of the breast shape and position is more difficult due to the mobile nature of the breast. Clinically, a smaller breast tends to flatten along the chest wall with the patient in a supine, arms up position, while a larger breast that remains pendulous in this position retains breast tissue that is can be very mobile. Larger breast size has been associated with increased acute toxicity such as breast edema [24]. Despite this tendency for higher breast error, the target coverage is well maintained (CTV D95 within 1% variation) as long as the patient’s posture setup is accurate (< 2° setup error). However, if the patient had a large (>2^o^) posture error, CTV D95 commonly decreased by ≥1%, and alignment with the breast tissue or the skin was not always sufficient to overcome the posture error, resulting in decreased target coverage. The patient’s body is not rigid; when the patient has a large posture error, 6D couch corrections can only be a compromise. The larger posture error in the initial setup, the more residual posture errors after couch corrections and the more dose errors will occur. Furthermore, the posture error may exacerbate the breast error, as seen in Fig. 2a, as the mobile breast tissue can align differently based on patient posture. Therefore, if >2^o^ posture error is identified on initial patient setup for daily treatment, we recommend correction of the posture by reset up the patient and repeat imaging until a posture error < 2^o^ is achieved.

At our center, the current standard practice of breast patient setup is to use the orthogonal KV images to align with the bony anatomy. Therefore, for treatment planning, we account for the relative motion between the bony and breast tissue through robust optimization with a 5-mm setup uncertainty. It is our expectation that an acceptable dose coverage can be achieved with bony alignment. Due to the robustness of our plan, no apparent CTV dose coverage difference was observed among the three types of alignment strategies. For future research, it would be interesting to investigate whether [1] it is acceptable to robust optimize the plan with a tighter setup uncertainty if CBCT is used for daily setup, and [2] a larger difference can be observed for robust optimized plans using a tighter setup uncertainty.

In the present study, we used different alignment methods for the verification CT to mimic different IGRT techniques. The image registration was conducted in RayStation using automatic registration on selected ROIs. We have to note that the image registration algorithms used in RayStation may differ from those used in the imaging system for patient setup and treatment delivery. The simulated surface alignment using the skin5mm rim may be especially different than the true optical-based surface alignment. Furthermore, others [25] have shown that setup accuracy for surface imaging is sensitive to the ROI selection. Therefore, it is essential to evaluate the limitations of a particular image guidance system and identify the differences in image registration algorithms before directly translating the current study results to clinical patient setups.

## Conclusion

A planning technique that utilizes robust optimization on CTVs with a 5-mm setup uncertainty and 3.5% range uncertainty successfully maintains acceptable target coverage with all alignment strategies (bony anatomy, breast tissue, and skin). Breast tissue and skin alignment maintained the breast target coverage marginally better than bony alignment. When aligning with bony structure, the CTV along the chest wall is the predominant area affected by under-dosing. The posture error is more influential to the target dose distribution than the breast error.

## Data Availability

The datasets used and/or analyzed during the current study are available from the corresponding author on reasonable request.
